# Postspinal anesthesia shivering in lower abdominal and lower limb surgeries: a randomized controlled comparison between paracetamol and dexamethasone

**DOI:** 10.1186/s12871-021-01483-7

**Published:** 2021-10-30

**Authors:** Ibrahim M. Esmat, Marwa M. Mohamed, Wail A. Abdelaal, Hazem M. El-Hariri, Tarek M. Ashoor

**Affiliations:** 1 Department of Anesthesia and Intensive Care, Faculty of Medicine, Ain-Shams University, Cairo, Egypt; 2 Community Medicine Department, National Research Centre, Cairo, Egypt

**Keywords:** Paracetamol, Dexamethasone, PSAS, Surgeries

## Abstract

**Background:**

Shivering is known to be a frequent complication in patients undergoing surgery under neuraxial anesthesia with incidence of 40–70%. Although many pharmacological agents have been used to treat or prevent postspinal anesthesia shivering (PSAS), the ideal treatment wasn’t found. This study evaluated the efficacy of paracetamol and dexamethasone to prevent PSAS in patients undergoing lower abdominal and lower limb surgeries.

**Methods:**

Three hundred patients scheduled for surgeries under spinal anesthesia (SA) were allocated into three equal groups to receive a single preoperative dose of oral paracetamol 1 g (P group), dexamethasone 8 mg intravenous infusion (IVI) in 100 ml normal saline (D group) or placebo (C group), 2 h preoperatively, in a randomized, double-blind trial. The primary endpoint was the incidence of clinically significant PSAS. Secondary endpoints included shivering score, the change in hemodynamics, adverse events (e.g., nausea, vomiting and pruritis) and patients` satisfaction.

**Results:**

Clinically significant PSAS was recorded as (15%) in P group, (40%) in D group and (77%) in C group (*P* < 0.001). The mean blood pressure values obtained over a 5-25 min observation period were significantly higher in the D group (*P* < 0.001). Core temperature 90 min after SA was significantly lower in the 3 groups compared to prespinal values (*P* < 0.001). Nausea, vomiting and pruritis were significantly higher in the C group (*P* < 0.001). P and D groups were superior to C group regarding the patients’ satisfaction score (*P* < 0.001).

**Conclusion:**

Paracetamol and dexamethasone were effective in prevention of PSAS in patients undergoing lower abdominal and lower limb surgeries compared to placebo controls.

**Trial registration:**

ClinicalTrials.gov Identifier: NCT03679065 / Registered 20 September 2018 - Retrospectively registered, http://www.ClinicalTrial.gov*.*

## Background

Spinal anesthesia (SA) is a safe anesthetic technique used for both elective and emergency operations. Shivering is known to be a frequent complication in patients undergoing surgery under neuraxial anesthesia with incidence of 40–70% [1]. SA inhibits tonic vasoconstriction and causes redistribution of core heat from the trunk (below the block level) to the peripheral tissues predisposing patients to hypothermia and shivering [2]. Postspinal anesthesia shivering (PSAS) is an involuntary, repetitive activity of skeletal muscles as a physiological response to core hypothermia to raise the metabolic heat production [3]. PSAS increases O_2_ consumption, CO_2_ production, plasma catecholamines and cardiac output. Shivering may interfere with the monitoring of ECG, blood pressure and oxygen saturation [4]. The mainstay of prophylaxis and treatment of PSAS remain pharmacological [1, 2, 4] due to inadequate control of central hypothermia by techniques based on physical principles (e.g., intravenous infusion (IVI) of warm fluids and forced air warmers) [5]. It appears logical to prevent PSAS rather than to treat it once it develops.

Paracetamol [Acetaminophen (ACT)] is an effective, safe synthetic non-opioid analgesic and antipyretic [6, 7]. Acetaminophen acts by inhibiting cyclooxygenase-mediated prostaglandin synthesis to decrease the hypothalamic temperature set point [7, 8]. Although the onset of action of IV paracetamol is much faster compared with its oral form and bioavailability is 63–84% of administered dose, there were no significant difference in overall efficacy between the two routes [6]. Rectal administration of acetaminophen proved to be effective for prevention of shivering in the therapeutic hypothermia [9].

Dexamethasone has an anti-inflammatory, analgesic effects and significantly improved the duration of sensory block in spinal anesthesia when added to hyperbaric bupivacaine [10]. The use of preoperative dexamethasone (8 mg), with a biologic half-life of 36–72 h, improved postdischarge quality of recovery [11] and decreased nausea, vomiting [11, 12], pain, and fatigue in the early postoperative period [11]. As it reduces the gradient between skin and body core temperature and modifies the inflammatory response, dexamethasone has been reported to be effective in reducing shivering after cardiac surgeries [13]. Dexamethasone may cause unpleasant symptoms following rapid IV injection [14].

The aim of this study was to evaluate the efficacy of a prophylactic dose of 1 g oral paracetamol tablet compared with a prophylactic dose of 8 mg dexamethasone IVI to prevent shivering in patients undergoing lower abdominal and lower limb surgeries under spinal anesthesia.

## Methods

### Patients

This double blind randomized controlled study was conducted between the 1st of March to the 31st of August 2018 at Ain-Shams University hospitals after approval by the institute ethics committee (FMASU R 49 / 2018) and was registered at ClinicalTrials.gov (NCT03679065). This study included 300 patients, of both sexes, aged 18–60 years, ASA I or II scheduled for elective lower abdominal or lower limb surgeries under spinal anesthesia and a written informed consent was obtained by every patient. “ The study protocol was performed in accordance to the ethical standards of the Declaration of Helsinki” [15].

Patients with generalized infection or localized infection at level of blockade, thyroid disease, cardiopulmonary disease, coagulation disorder, liver dysfunction, neurologic disease, psychological disorder, a known history of alcohol or substance abuse, a body mass index > 35 kg/m^2^ or refusal to participate in clinical research were excluded. Patients with an initial body temperature > 38 °C or < 36.5 °C, receiving medications likely to alter thermoregulation or vasodilators, required blood transfusion during surgery, operated for more than 120 min or with known allergy to the study medications were also excluded.

All patients were medically evaluated preoperatively. Preoperative fasting started 6 h before surgery and water intake was possible until 2 h before surgery.

### Randomization and blinding

Randomization of the patients was performed using a computer-generated random numbers concealed in sealed opaque envelopes and a nurse randomly chose the envelope that determined the group of assignment [16]. Patients were allocated into three equal groups (100 each) with 1:1:1 ratio according to shivering prophylaxis; Paracetamol (P) group:(*n* = 100) each patient received 1 g paracetamol tablet orally with sips of water and 100 ml 0.9% sodium chloride (normal saline [NS]) (IVI) over 15 min as a placebo for dexamethasone solution; dexamethasone (D) group: (*n* = 100) each patient received a placebo tablet identical to paracetamol tablet orally with sips of water and dexamethasone 8 mg ampoule diluted in 100 ml 0.9% NS IVI over 15 min; Control (C) group: (*n* = 100) each patient received a placebo tablet identical to paracetamol tablet orally with sips of water and 100 ml 0.9% NS IVI over 15 min as a placebo for dexamethasone solution 2 h preoperatively. The study medications including matching placebo tablets and fluids were given 2 h preoperatively.

Paracetamol was presented as (Novaldol® 1000 mg (1 g) tablets manufactured by SANOFI, Egypt) and dexamethasone was presented as (Dexamethasone Sodium Phosphate ampoules 8 mg in 2 ml, MUP Egypt). The study drugs were prepared by the hospital pharmacy and presented by a nurse who wasn’t involved in patients’ management. The attending anesthesiologists, surgeons and patients were blinded to the patients’ groups assignments. All follow up notes were recorded by anesthesia residents who followed-up the patients and were unaware of the nature of medications and were not involved in any other part of the study [16].

### Study protocol

The anesthetic management of the patients was standardized without any premedication. Before commencing spinal anesthesia, standard monitoring with a five-lead electrocardiogram (ECG), non-invasive blood pressure (NIBP), and peripheral arterial oxygenation (SPO_2_%) (Datex-Ohmeda S/5, GE Health Care, Finland) were established and all patients received IV lactated Ringer’s solution (at room temperature) at 10 ml/kg within 30 min and then at 6 ml/kg/h. All patients were given supplementary oxygen by a face mask at a rate of 5 L/min. Patients were covered with surgical drapes before and during the procedure and cotton blankets postoperatively. No patient received any active perioperative warming, as per our current practice standard of care. The operating and recovery rooms temperatures were maintained at 23–25 °C (measured by a wall thermometer) with approximately 60–70% humidity. The core body temperature was measured as the tympanic membrane temperature using an ear thermometer (ThermoScan IRT 4020, Braun, Kronberg, Germany) [17] and a core temperature below < 36.5 °C was considered hypothermia.

Following the guidelines for asepsis and antisepsis, subarachnoid anesthesia was instituted at either the L3–4 or L4–5 interspaces (midline approach) with the patient at sitting posture. A volume of (2.5–3.5 ml) (12. 5-17.5 mg) of hyperbaric bupivacaine (Marcaine® Spinal Heavy 0.5%; Sunny pivacaine, Manufactured by Sunny Pharmaceutical - Cairo - Egypt) was injected over 60 s using a 25 G Quincke spinal needle (Spinocaine, B. Braun Melsungen, Germany) to achieve a desirable level in accordance with the surgical procedure (considering height and weight of the patient). After completing the spinal block, withdrawal of the needle and covering the site of injection with a sterile gauze, the patient was positioned supine and a urinary catheter was inserted.

The time to peak sensory block level (min) (time taken from the end of injection to loss of pin prick sensation at a bilateral T4-T8 dermatomes), highest level of sensory blockade, duration of sensory blockade (2 segment regression time from highest level of sensory blockade), complete motor blockade (time taken from the end of injection to the development of grade 4 motor block, modified Bromage criteria [18]), duration of motor blockade (time required for motor blockade return to Bromage grade 1 from the time of onset of motor blockade), time to first analgesic rescue (min), changes produced in heart rate (HR), mean arterial pressure (MAP), peripheral arterial oxygen saturation (SPO_2_%), tympanic membrane temperature (T) and side effects were recorded at prespinal, 2, 5,10, 15, 20,25, 30, 40, 50, 60, 70, 80, 90 min after intrathecal injection. If patients did not develop sensory block up to T4-T8 and grade 4 motor block. The procedure was abandoned, general anesthesia was administered and those patients were excluded from the study.

After completion of the subarachnoid injection, the incidence and severity of shivering were recorded during the operation till 90 min after SA. Shivering severity was assessed with a four-point scale [19]:

1. None (Grade 0): no shivering noted on palpation of the masseter, neck, or chest wall
2. Mild (Grade 1): shivering localized to the neck and/or thorax only
3. Moderate (Grade 2): shivering involved gross movement of the upper extremities (in addition to neck and thorax)
4. Severe (Grade 3): shivering involved gross movements of the trunk and upper and lower extremities.

If the shivering grade was ≥2 after 15 min after completion of the subarachnoid injection, the prophylaxis was regarded as ineffective and 25 mg meperidine IV (diluted to 10 mL with NS) was slowly injected as a rescue agent. Parameters such as onset of shivering (the time in minutes at which shivering started after intrathecal injection), the patients` percentage at each grade of shivering among the three groups, response rate (number of cases in whom shivering ceased after administered meperidine in 10 min) and shivering recurrence were also recorded. PSAS was documented visually by 2 anesthesia residents who were un aware of the study group allocation. They were also carefully briefed on the shivering intensity assessment used in the study. Patients` satisfaction with shivering prophylaxis was recorded using a 7-point Likert verbal rating scale [20].

Side-effects like hypotension (MAP < 20% from baseline), bradycardia (HR < 50 beats/min), respiratory depression (respiratory rate ≤ 8/min or SPO_2_ ≤ 92%), nausea, vomiting and headache were recorded. Hypotension was treated with a bolus infusion of crystalloid (250 ml) and/or incremental dose of ephedrine 6 mg IV. Bradycardia was treated with atropine (0.01 mg/kg) IV. If a patient had both nausea and hypotension, incremental dose of ephedrine 6 mg IV was administered. If desaturation was detected, the patients were treated with supplemental oxygen via a nasal cannula at 3 l per minute (LPM) to keep SpO_2_ > 94%. if two or more emetic episodes occurred or nausea persisted for more than 10 min with normal BP or HR, metoclopramide 10 mg IV was given as rescue antiemetic. Patients with pruritus were treated with IV clemastine (Tavegyl^R^) (2 mg / ampoule). Sedation was checked every 15 min over 90 min and was assessed with a four-point scale as per Filos et al. [21].

The primary outcome was the incidence of clinically significant PSAS which required IV pethidine for treatment (Grade 2 (moderate) and Grade 3 (severe)) after the first 90 min (end point of the study) after the completion of the subarachnoid drug injection (start point of the study). The shivering score, the patients` satisfaction and the incidences of safety-related outcomes including nausea, vomiting, bradycardia, hypotension and sedation were the secondary outcomes.

### Statistical analysis

Based on a similar previous study, a minimal sample size of 19 cases in each group was required to provide *statistical significance* when the assumed differences between the three groups; group C, group P and group D were 33.3, 0.0 and 0.0% respectively [22] with setting the power = 0.80 and α = 0.017 [23] and using PASS 11th release [24]. The investigators recruited 100 cases in each group to compensate for patient’s dropout, protocol violation, further comparisons and finding possible side effects.

The collected data were coded tabulated and statistically analyzed using IBM SPSS statistics (Statistical Package for Social Sciences) software version 22.0, IBM Corp., Chicago, USA, 2013. Quantitative normally distributed data were described as mean ± SD (standard deviation) and were compared by ANOVA test and repeated measures analysis of variance (RMANOVA). In addition, quantitative non-normally distributed data were described as median and 1st& 3rd inter-quartile range and were compared by Kruskal Wallis test. On the other hand, qualitative data were described as number and percentage and were compared by Chi square test and Fisher’s Exact test for variables with small expected numbers. Over and above, post hoc testing was done using Bonferroni test with adjusting significant *P* value cut point to be < 0.017 in cases of paired groups comparisons and Log rank test was used to compare rates. The level of significance was considered significant if *P* value < 0.050, otherwise was non-significant. Effect size was calculated for the relative value of each medication over the other tested medications.

## Results

**Fig. 1:**
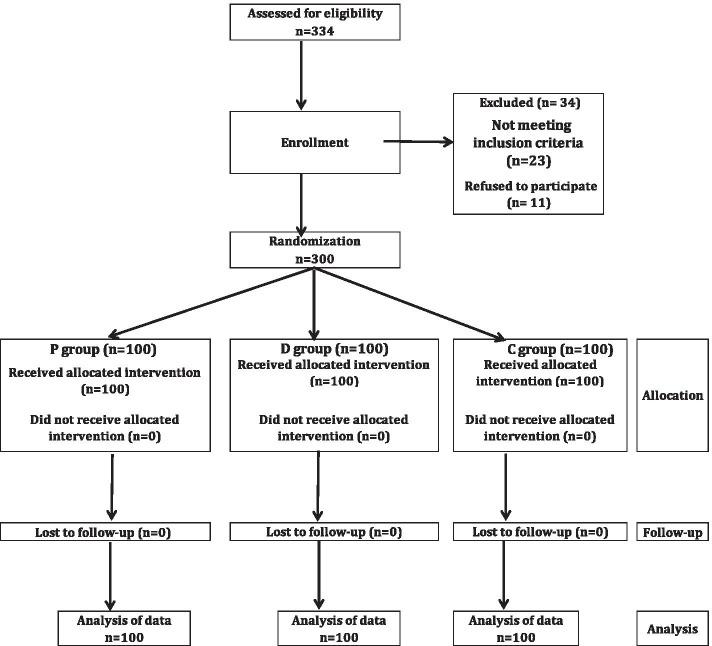
The study flow diagram

Three hundred thirty-four patients were assessed for eligibility; of those 300 patients completed the study and were randomized (100 patients for each group) between the 3 groups and their data were included in the final analysis. Thirty-four patients were excluded from this study on account of patients did not meet inclusion criteria (23 patients) and patients’ refusals (11 patients) (Fig. 1).

As shown in Table 1, no significant differences (*P* > 0.05) were noted between the 3 groups regarding age, sex, BMI, ASA status, bupivacaine dose, duration of surgery, types of operations and total IV fluid used. The time to peak sensory block level was statistically significant higher in the D group compared to P and C groups (*P* < 0.001) with no statistically significant differences between P and C groups (Table 2). Both of time to two-segments regression and time to first analgesic rescue were statistically significant lower in C group compared to the P and D groups with no statistically significant differences between P and D groups (*P* < 0.001, *P* < 0.001 respectively) (Table 2). The peak sensory block level, time to reach complete motor block and the duration of motor block were comparable between the three studied groups (*P* = 0.103, *P* = 0.069, *P* = 0.132 respectively) (Table 2).

**Table 1:** Demographic characteristics and perioperative data

Variables	P Group (***n*** = 100)	D Group (***n*** = 100)	C Group (***n*** = 100)	***P***-value
Age; years	44.4 (6.3)	44.0 (7.3)	45.1 (6.2)	^0.484
BMI; (kg/m^2^)	30.7 (2.1)	30.5 (2.4)	30.7 (2.3)	^0.769
Sex; (male/Female)	63/37	61/39	64/36	#0.905
ASA I/II	24/76	28/72	26/74	#0.812
Bupivacaine dose; (mg)	15.4 (1.1)	15.6 (1.1)	15.4 (1.1)	^0.271
Duration of surgery; (min)	99.6 (6.6)	98.9 (5.9)	98 (4.1)	^0.135
Type of operation; n, %				
• Inguinal hernia	28 (28.0%)	26 (26.0%)	29 (29.0%)	#0.999
• Vaginal Hysterectomy	12 (12.0%)	14 (14.0%)	14 (14.0%)	
• Vesico-vaginal fistula repair	11 (11.0%)	13 (13.0%)	12 (12.0%)	
• Myomectomy	9 (9.0%)	10 (10.0%)	7 (7.0%)	
• Internal fixation of tibial fractures	15 (15.0%)	13 (13.0%)	16 (16.0%)	
• Dynamic hip screw (DHS)	11 (11.0%)	11 (11.0%)	10 (10.0%)	
• Skin grafting	14 (14.0%)	13 (13.0%)	12 (12.0%)	
Total IV fluid used; ml	1958.0 (107.5)	1991.0 (110.2)	1978.0 (118.6)	^0.113

^ANOVA test. #Chi square test with post hoc Bonferroni test. *Significant

**Table 2:** Characteristics of neuraxial anesthesia techniques

Variables	P Group (***n*** = 100)	D Group (***n*** = 100)	C Group (***n*** = 100)	***P***-value	Effect size			
					Measures	P/D	P/C	D/C
Peak sensory block level; **(median [range])**	T5 (T4-T8)	T5 (T4-T8)	T6 (T4-T8)	§0.103	Not applicable			
Time to peak sensory block level; (min)	7.1 (0.6) a	4.7(0.6) b	6.9 (0.7) a	^< 0.001*	**M (SE)** **95% CI**	2.4 (0.1) 2.2–2.6	0.2 (0.1) 0.0–0.4	−2.2 (0.1) −2.4 – −2.0
Time to two-segments regression; (min)	78.9 (1.7) a	78.3 (2) a	66.5 (1.6) b	^< 0.001*	**M (SE)** **95% CI**	0.5 (0.3) 0.0–1.1	12.4 (0.2) 11.9–12.8	11.8 (0.3) 11.3–12.3
Time to reach complete motor block; min	8.7 (0.4)	8.7 (0.4)	8.8 (0.5)	^0.069	**M (SE)** **95% CI**	0.1 (0.1) −0.1–0.2	−0.1 (0.1) − 0.2–0.0	−0.1 (0.1) − 0.3–0.0
Duration of motor block; (min)	137.5 (2.4)	137.1 (2)	136.9 (2.1)	^0.132	**M (SE)** **95% CI**	0.4 (0.3) −0.2–1.0	0.6 (0.3) 0.0–1.3	0.3 (0.3) −0.3–0.8
Time to first analgesic rescue; (min)	339.9 (8.3) a	337.4 (8.8) a	217.6 (9.5) b	^< 0.001*	**M (SE)** **95% CI**	2.5 (1.2) 0.1–4.9	122.3 (1.3) 119.8–124.8	119.7 (1.3) 117.2–122.3

^ANOVA test. §Kruskal-Wallis test. Homogenous groups had the same symbol (a,b) by post hoc Bonferroni test. *Significant. CI: Confidence interval. Data were presented as mean and standard deviation or mean and standard error when applicable. *M* mean, *SE* standard error, *CI* confidence interval

**Fig. 2:**
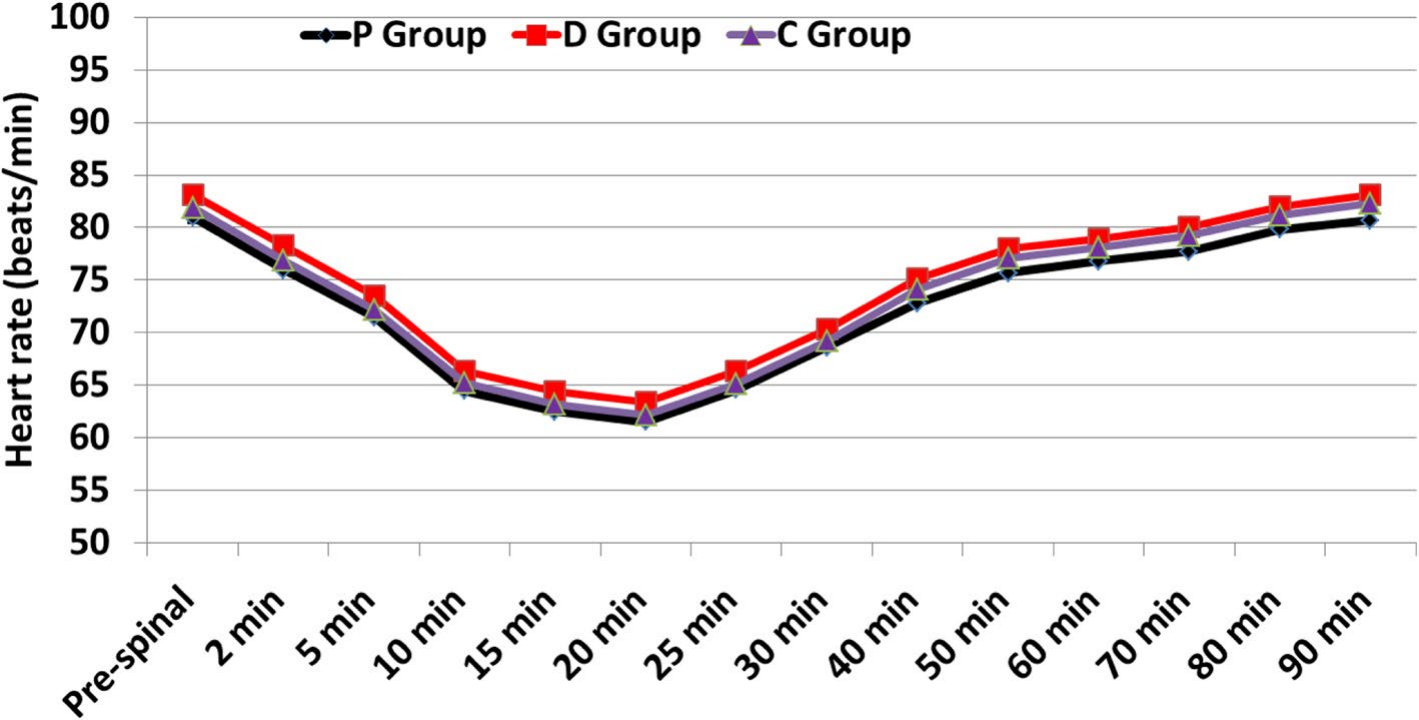
Changes in Heart rate (beats/min) with time

**Fig. 3:**
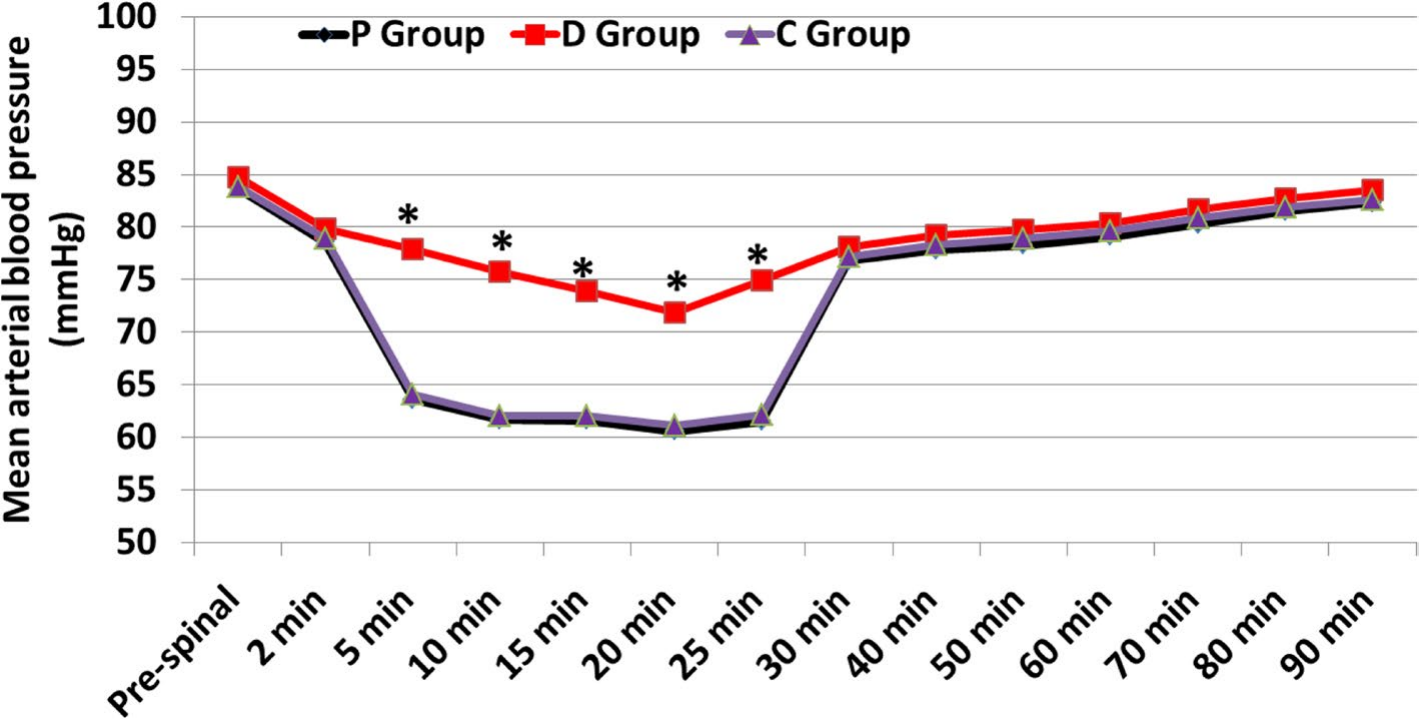
Changes in Mean arterial blood pressure (mmHg) with time. *Significant

The changes in heart rate values during the study period were comparable between the three studied groups (Fig. 2). The Repeated measured values of MBP (5 min, 10 min, 15 min, 20 min and 25 min after intrathecal injection) were statistically significant lower at P group than D group with mean difference (− 14.2. 95% CI: − 15.3–-13.1) while they were statistically significant higher in D group than in C group with mean difference (13.8. 95% CI: 12.6–15.0) (*P* < 0.001) with no statistically significant differences between P and C groups (Fig. 3). There were no significant differences between groups in the changes in SpO_2_ (%) values at any time point in the study (*P* > 0.05).

**Fig. 4:**
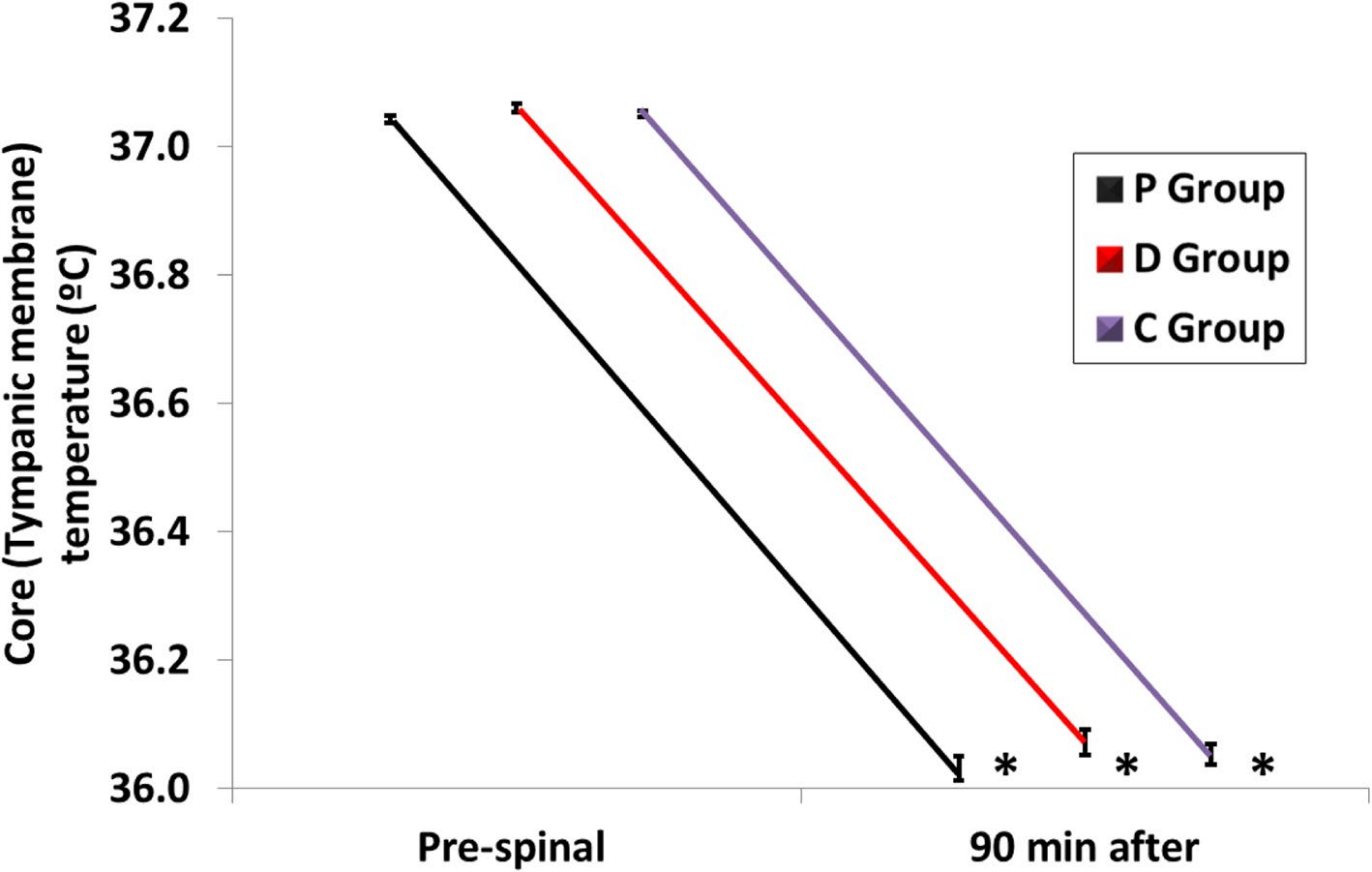
Changes in Core (Tympanic membrane) Temperature (C°) 90 min after intrathecal injection when compared with the pre-spinal values. *Significant. RMANOVA test was used

**Fig. 5:**
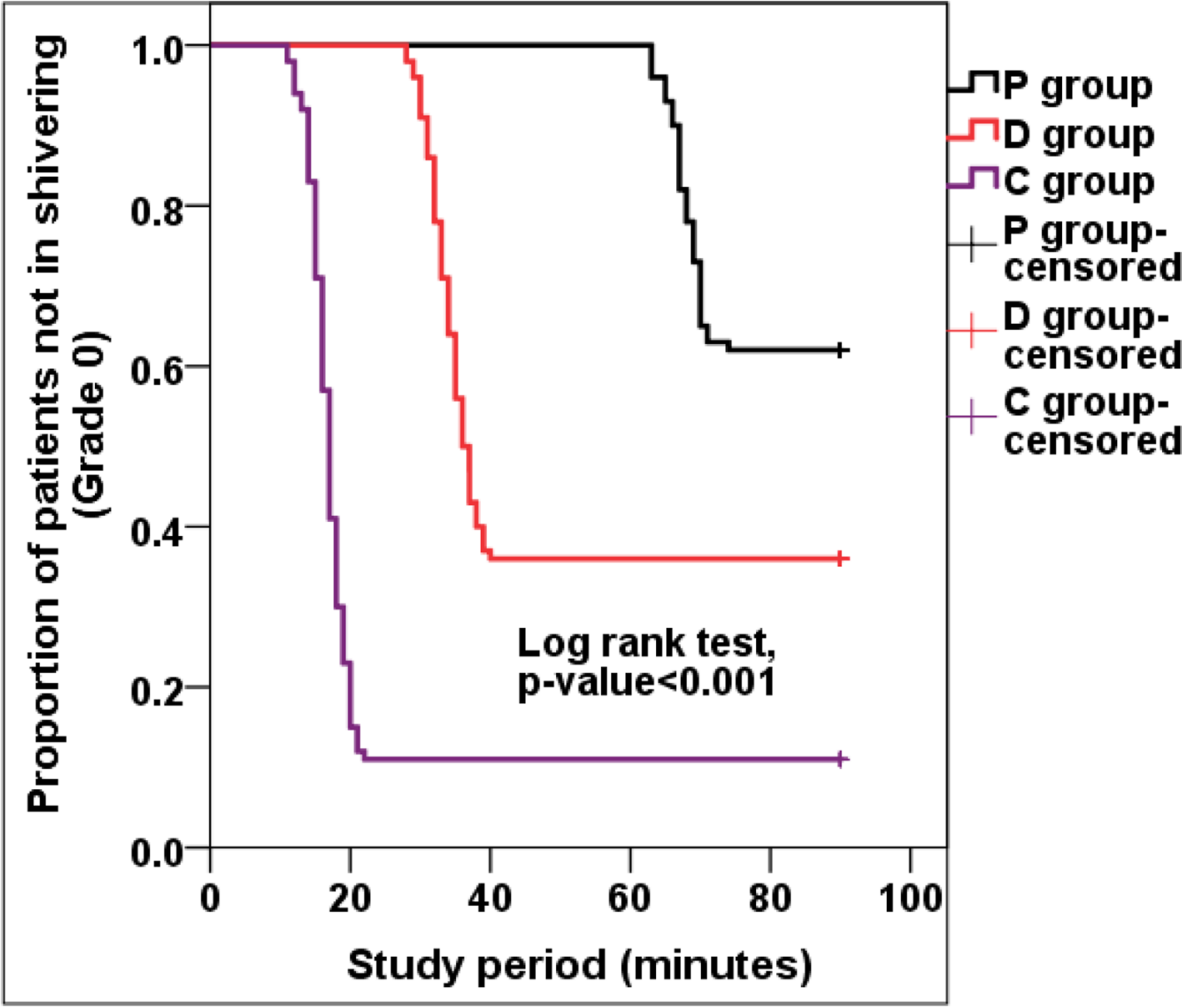
Kaplan-Meier plot of the percentage of patients in each group not in shivering

**Fig. 6:**
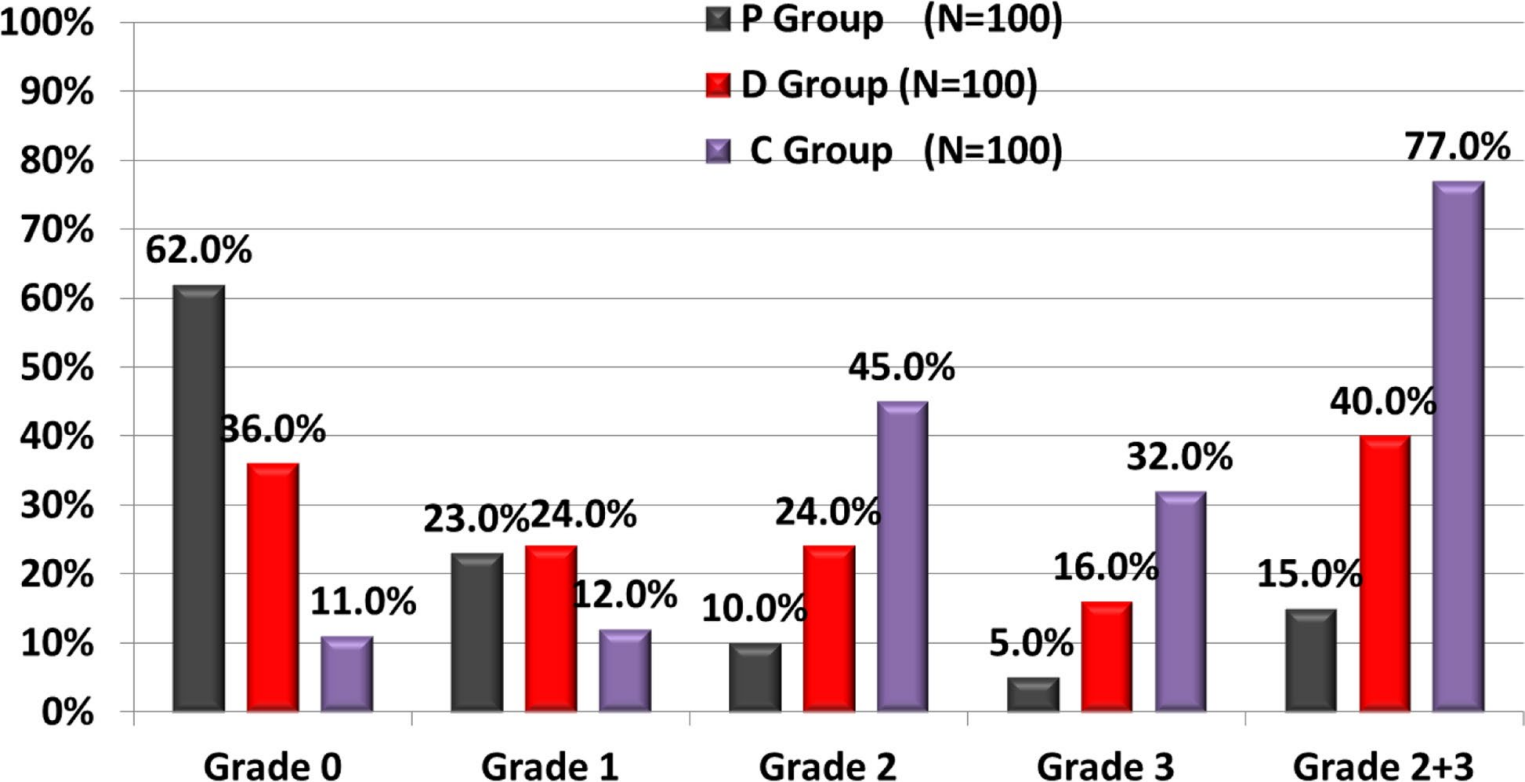
The percentage of patients at each grade of shivering

The 90-min after intrathecal injection core temperature was statistically significant lower in P, D and C groups compared to prespinal core temperature in the 3 groups (*P* < 0.001) with no statistically significant differences between P, D and C groups at any time point of the study period (Fig. 4). The incidence of shivering was significantly higher in C group compared to P and D groups with statistically significant differences between P and D groups (*P* < 0.001) while the onset of shivering was significantly lower in C group compared to P and D groups with statistically significant differences between P and D groups (*P* < 0.001) (Table 3) (Fig. 5). Clinically significant shivering (shivering grade ≥ 2) was recorded in 15/100 patients (15.0%) in P group, 40/100 (40.0%) in D group and 77/100 (76.0%) in C group (*P* < 0.001) (Table 3) (Fig. 6). The need for anti-shivering treatment showed statistically significant differences between the studied groups, was the least frequent in P group (15.0%) with relative rate when compared to D group (0.38; 95% CI: 0.22–0.63) and relative rate when compared to C group (0.19; 95% CI: 0.12–0.31), followed in frequency by D group (40.0%) with relative rate when compared to C group (0.52; 95% CI: 0.40–0.68), and was the most frequent in C group (77.0%). The mean dose of IV meperidine required to treat shivering was significantly higher in C group compared to P and D groups with statistically significant differences between P and D groups (*P* < 0.001) (Table 3). The response rate (complete cessation of shivering activity within 10 min of one dose of meperidine 25 mg) was 100.0%% in P group, 70.0% in D group and 40.3% in C group (*P* < 0.001). The recurrence of shivering was significantly higher in D and C groups compared to P group with statistically significant differences between D and C groups (*P* < 0.001) (Table 3).

**Table 3:** Shivering profile in the studied groups

Variables	P Group (***n*** = 100)	D Group (***n*** = 100)	C Group (***n*** = 100)	***P***-value	Effect size			
					Measures	P/D	P/C	D/C
Incidence of shivering; n, %	38 (38.0%) a	64 (64.0%) b	89 (89.0%) c	#< 0.001*	**RR** **95% CI**	0.59 (0.44–0.79)	0.43 (0.33–0.55)	0.72 (0.61–0.85)
Shivering grade; n, %								
• 0	62 (62.0%) a	36 (36.0%) b	11 (11.0%) c	#< 0.001*	Not applicable			
• I	23 (23.0%) a	24 (24.0%) a	12 (12.0%) a					
• II	10 (10.0%) a	24 (24.0%) b	45 (45.0%) c					
• III	5 (5.0%) a	16 (16).0% b	32 (32.0%) c					
Patients in need for anti-shivering treatment (Grades 2 and 3); n, %	15 (15.0%) a	40 (40.0%) b	77 (77.0%) c	#< 0.001*	**RR** **95% CI**	0.38 (0.22–0.63)	0.19 (0.12–0.31)	0.52 (0.40–0.68)
Onset of Shivering; (min)	67.7 (2.5) a	33.9 (2.9) b	16.6 (2.4) c	^< 0.001*	**M (SE)** **95% CI**	33.8 (0.6) 32.7–35.0	51.2 (0.5) 50.2–52.1	17.3 (0.4) 16.5–18.2
Meperidine dose; (mg)	25.0 (0) a	32.5 (11.6) b	39.9 (12.3) c	^< 0.001*	**M (SE)** **95% CI**	−7.5 (3) −13.5–-1.5	−14.9 (3.2) −21.3–-8.6	−7.4 (2.4) −12.1–-2.8
Response rate; n, %	15 (100.0%) a	28 (70.0%) b	31 (40.3%) c	#< 0.001*	**RR** **(95% CI)**	Not applicable	Not applicable	1.74 (1.24–2.44)
Recurrence; n, %	0 (0.0%) a	12 (30.0%) b	46 (59.7%) c	#< 0.001*	**RR** **95% CI**	Not applicable	Not applicable	0.5 (0.30–0.83)

^ANOVA test, #Chi square test. Homogenous groups had the same symbol (a, b, c) by post hoc Bonferroni test. *Significant. *RR* Relative risk, *CI* Confidence interval, *M* Mean, *SE* Standard error

The incidence of hypotension, the percentage of patients who required ephedrine to treat post-spinal anesthesia (PSA) hypotension and the mean dose of IV ephedrine required were significantly higher in P and C groups compared to D group with no statistically significant differences between P and C groups (*P* < 0.001, *P* < 0.001, *P* < 0.001 respectively) (Table 4). Twenty-six patients (24.0%) complained from pruritus in C group, while 7 and 9 patients (2.0 and 3.0%) complained from pruritus in P and D groups respectively (*P* < 0.001) (Table 4). The number of nausea episodes, vomiting episodes, and rescue antiemetic usage were statistically significant lower in the P and D groups compared to C group (*P* < 0.001, *P* < 0.001, *P* < 0.001 respectively) with no statistically significant differences between the P and D groups (Table 4). Post-intervention sedation was grade I in all study groups (Table 4). Higher patients` satisfaction mean scores about shivering prophylaxis were recorded in P and D groups compared with C group with no statistically significant differences between P and D groups (*P* < 0.001) (Table 4).

**Table 4:** Adverse effects, administered drugs for treatment and patients` satisfaction score

Variables	P Group (***n*** = 100)	D Group (***n*** = 100)	C Group (***n*** = 100)	***P***-value	Effect size			
					Measures	P/D	P/C	D/C
Bradycardia; n, %	10 (10.0%)	6 (6.0%)	8 (8.0%)	#0.581	**RR** **95% CI**	1.67 0.63–4.41	1.25 0.51–3.04	0.75 0.27–2.08
Hypotension; n, %	28 (28.0%) a	8 (8.0%) b	25 (25.0%) a	#< 0.001*	**RR** **95% CI**	3.50 1.68–7.30	1.12 0.71–1.78	0.32 0.15–0.67
Patients in need for ephedrine; n, %	75 (75.0%) a	36 (36.0%) b	72 (72.0%) a	#< 0.001*	**RR** **95% CI**	2.08 1.57–2.77	1.04 0.88–1.23	0.50 0.37–0.67
Ephedrine dose; (mg)	23.7 (3.4.0%) a	13.9 (3.2.0%) b	22.5 (3.8.0%) a	^< 0.001*	**M (SE)** **95% CI**	9.9 (0.7) 8.5–11.2	1.3 (0.6) 0.1–2.4	−8.6 (0.7) −10.1–-7.1
Pruritus; n, %	7 (7.0%) a	9 (9.0%) a	26 (26.0%) b	#< 0.001*	**RR** **95% CI**	0.78 0.30–2.01	0.27 0.12–0.59	0.35 0.17–0.70
Nausea; n, %	9 (9.0%) a	6 (6.0%) a	27 (27.0%) b	#< 0.001*	**RR** **95% CI**	1.50 0.55–4.06	0.33 0.17–0.67	0.22 0.10–0.51
Vomiting; n, %	5 (5.0%) a	3 (3.0%) a	16 (1.0%) b	#< 0.001*	**RR** **95% CI**	1.67 0.41–6.79	0.31 0.12–0.82	0.19 0.06–0.62
Patients in need for rescue antiemetic; n, %	6 (6.0%) a	3 (3.0%) a	24 (24.0%) b	#< 0.001*	**RR** **95% CI**	2.00 0.51–7.78	0.25 0.11–0.59	0.13 0.04–0.40
Sedation Grade I; n, %	100 (100.0%)	100 (100.0%)	100 (100.0%)	Not applicable				
Headache; n, %	5 (5.0%)	7 (7.0%)	8 (8.0%)	#0.687	**RR** **95% CI**	0.71 0.23–2.18	0.63 0.21–1.84	0.88 0.33–2.32
Patients` Satisfaction Score	5.1 (0.7) a	5.0 (0.8) a	2.5 (0.6) b	^< 0.001*	**M (SE)** **95% CI**	0.1 (0.1) −0.1–0.3	2.6 (0.1) 2.4–2.8	2.5 (0.1) 2.3–2.7

^ANOVA test and #Chi square test. Homogenous groups had the same symbol (a,b,c) by post hoc Bonferroni test. *Significant. *RR* Relative risk, *CI* Confidence interval, *M* Mean, *SE* Standard error. Post intervention sedation was Grade I in all study groups

## Discussion

The analysis of results across groups by primary outcome measure of clinically significant shivering (shivering grade ≥ 2) showed clinically and statistically significant superiority of both a prophylactic dose of oral paracetamol 1 g tablet and a prophylactic dose of dexamethasone 8 mg IVI compared with placebo controls for prophylaxis of shivering in patients undergoing lower abdominal and lower limb surgeries under spinal anesthesia. Moreover, patients in P and D groups showed significant longer time to two segments regression, increased time to first analgesic rescue, reduced incidence of nausea, vomiting and pruritus compared to C group with no significant differences between P and D groups.

The potential additive or synergistic effects of prophylactic administration of nonpharmacological and pharmacological methods to minimize core temperature loss are favored to prevent PSAS. The American Society of Anesthesiologists (ASA) guidelines suggest that patients should have their core temperatures maintained at ≥36.5 °C using forced-air warming devices and meperidine received the highest validation [25]. However, meperidine has adverse events such as nausea, vomiting, pruritus, sedation, hypotension, bradycardia and respiratory depression [4]. These data encouraged the investigators to seek for medications with minimal side effects that could replace the use of IV meperidine for prevention of PSAS.

The time frame of 90 min of the study was chosen based on studying the three main mechanisms of spinal anesthesia causing core hypothermia which reach their maximal effects during the first 30–60 min after intrathecal injection and patients should be monitored, actively warmed and receive antishivering treatment if needed [26]. Moreover, the dose and the timing of administration of 1 g oral paracetamol 2 h preoperatively was selected to achieve adequate shiver control according to the Columbia anti-shivering protocol and Raffa et al., who reported a significantly prolonged mean time to peak plasma concentration of oral paracetamol when co-administered with morphine infusion compared to oral paracetamol alone [8, 27]. Over and above, Fenlon et al., had advocated clinicians to avoid the additional costs and risks attached to the IV paracetamol due to the perceived benefits of IV paracetamol over oral are less than may be imagined and unlikely to significantly alter the patient’s perception of pain after surgery [6]. While the timing of administration of a preoperative single dose of 8 mg dexamethasone IVI was selected based on data obtained from a previous study [14] which validated the optimal effective dose [11, 14] in the prevention of postoperative nausea or vomiting after laparoscopic cholecystectomy (LC).

The three groups in terms of basic and demographic variables of age, sex, body mass index, ASA, bupivacaine dose, duration of surgery, types of operations and total IV fluid used weren’t significantly different (Table 1). The confounding effect of these factors in this study was neutralized and the observed differences were likely due to the taken drug.

There was a significant longer time to two segments regression and increased time to first analgesic rescue in P group compared to C group. Different mechanisms for the antinociceptive action of paracetamol, including cannabinoid receptor type 1(CB1) agonistic activity, reinforcement of descending serotonergic inhibitory pain pathways and prostaglandin synthesis inhibition [7, 9, 28]. Furthermore, plethora of studies supported results of this study in patients under spinal anesthesia receiving a prophylactic dose of 1 g oral paracetamol tablet regarding prolonged duration of sensory loss [28] and increased time to first analgesic rescue [6, 7, 9, 28]. On the other hand, our results coincided with the studies evaluating the efficacy of dexamethasone whether IV [11, 14, 29] or intrathecally [4, 10] when it was added to bupivacaine in decreasing the mean time to peak sensory block level and increasing both the time to two-segment regression and the mean time to requirement of the first rescue analgesia. There was *a* significant *attenuation of* PSA hypotension in D group which could be explained by the beneficial effects of increasing peripheral vascular resistance (PVR) by a variety of mechanisms and its anti 5HT_3_ effects which might influence the Bezold Jarish reflex (BJR) [16].

The intense vasodilation below the level of the spinal blockade will result in loss of thermoregulation and core hypothermia regardless of the other factors (e.g., ambient temperature and duration of surgery). Furthermore, vasoconstriction and shivering are thermoregulatory mechanisms that are required to increase core body temperature in patients with core hypothermia and are restricted to the upper body during spinal anesthesia [26]. Although a high level of spinal blockade [30] was achieved in this study (Block height) (dermatome level) (T_5_-T_6_) and a significant relationship between prespinal and 90 min after intrathecal injection core body temperature readings in the 3 study groups was documented by the investigators, a clinically significant shivering was significantly lower in P and D groups compared to C group. This could be explained due to paracetamol acts by inhibiting cyclooxygenase-mediated prostaglandin synthesis to lower the hypothalamic set point [8] while dexamethasone decreases the temperature gradient between core and skin temperatures via its anti-inflammatory action and inhibition of the release of vasoconstrictor and pyrogenic cytokines [13].

Consistent with our results, Kinjo et al., assumed that the administered acetaminophen prevented postoperative shivering by suppressing the postoperative increase in the core body temperature set point, rather than lowering the threshold for shivering [31]. In addition, Honasoge et al., also reported that shivering was effectively suppressed using rectal administration of buspirone and acetaminophen in the setting of therapeutic hypothermia for cardiac arrest [32]. Concomitant with our results, previous studies [11, 13] reported that dexamethasone significantly reduced the incidence of postoperative shivering. Moreover, Moeen et al., reported that intrathecal dexamethasone was as effective as intrathecal meperidine in attenuation of PSAS compared to placebo in patients scheduled for prostate surgery under spinal anesthesia with less side effects [4]. Over and above, previous studies supported our results regarding the mean dose of IV meperidine, used to treat clinically significant shivering, which was significantly reduced in P and D groups compared to C group [4, 9, 22].

Saglam et al., supported the results of this study regarding the lower incidence of pruritis in P group and they speculated that paracetamol attenuates the scratching behavior with its higher doses [33] while Apfel et al., attributed the antiemetic effect of acetaminophen to the reduction of postsurgical pain and not to the reduction of postoperative opioid consumption [34]. Over and above, the lack of an antipruritic effect of dexamethasone is disappointing in light of its antiemetic and anti-inflammatory properties [35]. Banihashem et al., however, documented that the severity of pruritus was significantly less in the dexamethasone treatment group than in the control group which could support our results [36]. On the other hand, the higher incidence of pruritus, nausea and vomiting in patients of C group could be due to increased consumption of IV meperidine possessing potential side effects such as pruritus, nausea and vomiting [4]. Also, the investigators reported increased consumption of IV ephedrine in patients of C group which may account for possibility of systemic hypersensitivity reactions [37].

Chen et al., reported that paracetamol had a direct anxiolytic effect through action on mood mechanisms [38]. Furthermore, previous investigators have reported that dexamethasone has a mood-altering effect and ability to produce a general sense of well-being due to a primary central nervous system effect of steroids [11]. Considering *all* the *above*-*mentioned advantages* of paracetamol and dexamethasone, this could explain the higher satisfaction score in P and D groups and reduced side effects of SA compared to C group.

This study had several limitations. First, this study was carried out at a single center. However, the investigators believed that the randomized double-blind design and the effect size estimation decreased the possibility of bias and the relatively large sample size achieved significant differences in the adverse events encountered. Second, the results of this study didn’t include patients with cardiovascular instability, endoscopic urosurgical procedures, invasive operations associated with increased blood loss nor major fluid shift. Despite that, the research team demonstrated that paracetamol and dexamethasone attenuated the effect of high levels of spinal blockade which decreased the core temperature threshold for shivering [30]. Moreover, dexamethasone demonstrated beneficial responses to avoid the methods that are used to avoid the PSA hypotension (e.g., volume loading or vasopressor administration) which may add the risk of hypervolemia and/or myocardial ischemia [16]. Third, this study lacked warmed intravenous fluids. In our hospitals, the use of warmed intravenous fluids is reserved for emergency operations and operations suspected to be of prolonged duration. However, the investigators believed that the use of paracetamol and dexamethasone in this study provided a simple, proved efficacy as antiemetic, less adverse effects and cost-effective management protocol for PSAS that could be easily applied in resource-limited areas. Fourth, the research team did not use a standard anti-shivering drug like meperidine as a control group as we considered it the rescue medication for all patients if paracetamol and dexamethasone failed to prevent distressing shivering. Fifth, glycemic profile in the hours after dexamethasone administration and the rate of infection should have been documented. Sixth, difficulties with patients` satisfaction assessment [20] might be invincible because perceptions of quality of care are subjective. In the future studies, the reasons for dissatisfaction should be mentioned for both satisfied and dissatisfied patients, to give a validity check of the global satisfaction questions for both groups [39].

The use of paracetamol or dexamethasone in this study *offered* several *notable benefits without eminent side effects.* Aside from its well-established analgesic and antipyretic effects, a prophylactic dose of 1 g oral paracetamol tablet proved efficacy in prevention of PSAS. As the dictum says, “prevention is better than cure,” it holds true for shivering also and it should be practiced. Since the suggested prevention of PSAS was efficacious, simple, inexpensive, available in many of operating rooms (ORs) and relatively safe, the research team strongly suggests trying the use of paracetamol and dexamethasone to prevent PSAS in patients exposed to risk factors [30].

## Conclusion

Paracetamol and dexamethasone were effective in prevention of PSAS in patients undergoing lower abdominal and lower limb surgeries under spinal anesthesia compared to placebo controls. Our results indicated that decreasing the threshold of shivering might be more important than increasing the core temperature in terms of preventing PSAS with less adverse events.

## Data Availability

The data that support the findings of this study were available from Ain-Shams University Hospitals and they were not publicly available. Data were however available from the authors upon reasonable request and with permission of Ain-Shams University.

## Abbreviations

5HT_3_: 5-Hydroxytryptamine 3
ALT: Alanine Aminotransferase
ASA: American Society of Anesthesiologists
AST: Aspartate Aminotransferase
BJR: Bezold Jarish reflex
DBP: Diastolic blood pressure
ECG: Electrocardiogram
HR: Heart rate
IQR: Interquartile range
IV: Intravenous
MBP: Mean blood pressure
NIBP: Non-invasive blood pressure
NS: Normal saline
ORs: Operating rooms
PSA: Postspinal anesthesia
PSAS: Postspinal anesthesia shivering
SA: Spinal anesthesia
SBP: Systolic blood pressure
SD: Standard deviation
SNOSE: Sequentially numbered, opaque sealed envelopes
